# Exploring the Dynamic Crosstalk between the Immune System and Genetics in Gastrointestinal Stromal Tumors

**DOI:** 10.3390/cancers15010216

**Published:** 2022-12-29

**Authors:** Alessandra Dimino, Chiara Brando, Laura Algeri, Valerio Gristina, Erika Pedone, Marta Peri, Alessandro Perez, Ida De Luca, Roberta Sciacchitano, Luigi Magrin, Tancredi Didier Bazan Russo, Marco Bono, Nadia Barraco, Silvia Contino, Maria La Mantia, Antonio Galvano, Giuseppe Badalamenti, Antonio Russo, Viviana Bazan, Lorena Incorvaia

**Affiliations:** 1 Department of Surgical, Oncological and Oral Sciences, Section of Medical Oncology, University of Palermo, 90127 Palermo, Italy; 2 Department of Biomedicine, Neuroscience and Advanced Diagnostics (Bind), Section of Medical Oncology, University of Palermo, 90127 Palermo, Italy

**Keywords:** sarcomas, GIST, target therapy, immunotherapy, immune checkpoints, tumor microenvironment, immune system

## Abstract

**Simple Summary:**

The effect of genetic alteration on the prognosis of patients affected by GIST has been extensively demonstrated. Unfortunately, not all GISTs could benefit from targeted therapies, underlining the need to deeply understand other predictive mechanisms. The link between immune checkpoints (especially PD-L1 expression), the tumor microenvironment, and the clinical behavior of GIST with different driver mutations is under investigation and represents an intriguing research field that could lead to improved prognostication in GIST.

**Abstract:**

Gastrointestinal Stromal Tumors (GISTs) represent a paradigmatic model of oncogene addiction. Despite the well-known impact of the mutational status on clinical outcomes, we need to expand our knowledge to other factors that influence behavior heterogeneity in GIST patients. A growing body of studies has revealed that the tumor microenvironment (TME), mostly populated by tumor-associated macrophages (TAMs) and lymphocytes (TILs), and stromal differentiation (SD) have a significant impact on prognosis and response to treatment. Interestingly, even though the current knowledge of the role of immune response in this setting is still limited, recent pre-clinical and clinical data have highlighted the relevance of the TME in GISTs, with possible implications for clinical practice in the near future. Moreover, the expression of immune checkpoints, such as PD-L1, PD-1, and CTLA-4, and their relationship to the clinical phenotype in GIST are emerging as potential prognostic biomarkers. Looking forward, these variables related to the underlying tumoral microenvironment in GIST, though limited to still-ongoing trials, might lead to the potential use of immunotherapy, alone or in combination with targeted therapy, in advanced TKI-refractory GISTs. This review aims to deepen understanding of the potential link between mutational status and the immune microenvironment in GIST.

## 1. Introduction

In the late 1990s, the therapeutic approach for patients with advanced gastrointestinal stromal tumors (GISTs) was dramatically revolutionized by the development of targeted therapies that completely redesigned the clinical history of this neoplasm. In this clinical setting, the administration of imatinib mesylate (IM) and several other multi-kinase inhibitors has been undoubtedly associated with improved outcomes for patients [1]. GISTs’ sensitivity to targeted therapies strictly relies on the presence of pathogenic alterations occurring mainly on the tyrosine kinase receptor (c-KIT) and platelet-derived growth factor receptor A (PDGFRA) genes [2].

The effect of genetic alteration on the prognosis of patients affected by GIST has been extensively demonstrated. Exon 11 c-KIT mutations are indicators of poor prognosis, while PDGFRA-driven mutation is usually associated with a favorable prognosis [3]. Unfortunately, not all GISTs will benefit from imatinib (IM) administration due to the presence of intrinsic primary resistance mutations (i.e., PDGFRA exon 18 D842V) or the occurrence of secondary resistance mutations to standard tyrosine kinase inhibitors (TKIs), leading to an unavoidable lack of clinical benefit in the later lines [4].

Interestingly, even though the current knowledge of the role of immune response in this setting is still limited, recent pre-clinical and clinical data highlighted the relevance of the tumor microenvironment (TME) in GISTs beyond the known impact of mutational signature [5]. This is well illustrated by the interesting fact that GISTs are frequently associated with gastric adenocarcinomas. These are almost exclusively low-risk, spindle-cell micro-GISTs (<2 cm), and their size shows an inverse correlation with distance from the adenocarcinoma, suggesting that the adenocarcinoma not only plays a role in their development but may also control the biological behavior of these GISTs, probably through modulation of their TME, including the immune environment [6]. Interestingly, the TME of primary and metastatic GISTs is populated by several immune cell types driving the immune-modulated tumor response. For instance, tumor-associated macrophages (TAMs), M2 macrophages and T-regulatory cells (Tregs) in particular, seem to preferentially populate metastatic sites, guiding strong immunosuppressive behavior [7,8]. Contrarily, the presence of NK cells localized preferentially within the fibrous stroma surrounding tumor cells was significantly associated with a low mitotic index and, along with CD3+ T cells, correlated with a reduced relapse rate and improved prognosis in untreated metastatic GISTs [9]. Furthermore, not only the gene but also the type of pathogenic variant can be related to a stronger immune-related behavior of GISTs [5]. Indeed, tumors harboring the PDGFRA exon 18 D842V pathogenetic alteration appeared to be enriched in immune cells, mainly CD8+ T cells, as compared to non-D842V ones [10]. Moreover, the expression of immune checkpoints, such as PD-L1, PD-1, and CTLA-4, and their relationship to the clinical phenotype in GIST are emerging as potential prognostic biomarkers and could lead to improved prognostication in GIST, traditionally based on mitotic indices, tumor location, and tumor size [5,11,12,13].

Looking forward, these variables related to the underlying tumoral microenvironment in GIST, even if limited to still-ongoing trials, might lead to the potential use of immunotherapy, alone or in combination with targeted therapy, in advanced TKI-refractory GISTs. This review aims to deepen the potential link between mutational status and the immune microenvironment in GIST.

## 2. Oncogenic Activation of KIT/PDGFRA Receptor Tyrosine Kinases: Setting the Stage for the “Oncogene Addiction” Model in GIST

GISTs are a subgroup of rare mesenchymal tumors with variable clinical behavior and reported incidence of from 0.4 to 2 cases per 100,000 per year [14]. GISTs can arise from any part of the gastrointestinal tract (GI), most frequently from the stomach (~60%) and small intestine (~30%); less frequently from the colon, rectum, and esophagus; and rarely outside the GI tract (mesentery, omentum, and retroperitoneum) [15].

Approximately 80–90% of GISTs are characterized by the presence of mutually exclusive driver mutations in either c-KIT or PDGFRA [16,17]. KIT/PDGFR-A wild-type (WT) GISTs, accounting for 10% and 85% of cases in adults and children, respectively, could carry other targetable driver mutations, more frequently in the BRAF, RAS, NTRK, neurofibromin 1 (NF1) genes or succinate dehydrogenase complex (SDH) genes [18]. The SDH-deficient subtype is most common in the pediatric population, whereas NF1-mutant GISTs are typically implicated in hereditary syndromes [19]. Indeed, in a minority of patients, GIST onset can be linked to Type 1 neurofibromatosis (NF1), characterized by a germline mutation of the NF1 gene, and Carney–Stratakis syndrome, marked by a germline pathogenic variant of one of the subunit genes of the SDH enzyme complex, linked to hypermethylation of the SDHC gene [20].

KIT and PDGRFA driver mutations represent not only key diagnostic markers but also prognostic factors and predictive biomarkers of effectiveness of molecular targeted therapy, and they have transformed GISTs into the known model of oncogene addiction [20]. IM, as a first-line treatment, improves metastatic, recurrent, and/or unresectable GIST patients’ survival. GISTs with a c-KIT driver mutation, accounting for 90% of adult GISTs, especially in exon 11 (50–77%), are highly sensitive to the standard dose of IM. GISTs with c-KIT exon 9 mutation are, instead, more sensitive to an increased dose of imatinib of 800 mg/die [14,18,21]. For patients with metastatic GIST progressing on IM, sunitinib, regorafenib, and ripretinib as second-, third- and fourth-line treatments, respectively, clinically improve objective response and PFS [22,23,24,25,26,27,28]. For PDGFRA exon 18 D842V-mutated GIST, which results in primary resistance to IM, avapritinib is a valid therapeutic option [29].

Although IM and other TKIs have profoundly changed the therapeutic landscape for patients with metastatic GISTs, the occurrence of primary and secondary resistance mechanisms is still a major clinical challenge, with the treatment of patients with PDGFRA exon 18 D842V mutations or KIT/PDGFRA WT GISTs still being controversial [30,31,32,33].

Despite most GISTs harboring KIT or PDGFRA mutations being highly sensitive to first-line imatinib, progression-free survival (PFS) and recurrence free-survival (RFS) can vary widely in these subsets of patients. In particular, the variable clinical outcomes in GIST patients with tumors harboring the same mutational status in terms of the type and gene location of mutations highlights the potential impact of different, intrinsic, immunological features on clinical outcomes.

## 3. The Immune System Is Not Far from Mutated GIST Cancer Cells: Is There a Link?

### 3.1. Tumor-Infiltrating Immune Cells in GISTs

**Figure 1:**
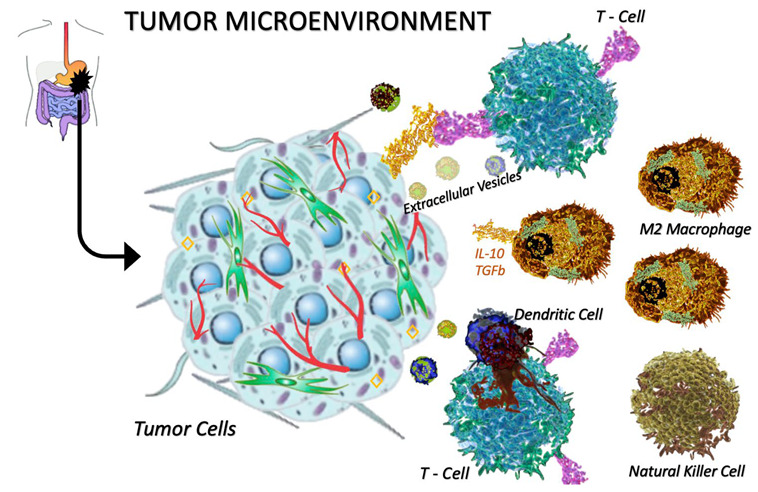
The tumor microenvironment population in cancer. The tumor microenvironment (TME) is populated by several immune cell types. In this figure, the following are represented: T-cells, M2 macrophages, dendritic cells, and natural killer cells. The M2 macrophages are characterized by their high expression of cytokines such as IL-10 and TGFb; thanks to these cytokines, the M2 macrophages promote an immunosuppressive environment.

The TME is a complicated system in which cancer cells coexist with other cells, such as tumor-associated fibroblasts, endothelial cells, and immune cells, which seem to play an important role during all the steps of tumor development and growth [34,35]. Inevitably, tumor cells become able to escape the immune response by stimulating an immune-suppressive TME [34] (Figure 1).

The presence of both innate and adaptive immune cells in solid tumors and their correlation with the clinical outcome of patients have been widely demonstrated [34]. A growing body of studies has revealed that the TME of GISTs, mostly populated by TAMs and tumor-infiltrating lymphocytes (TILs) [7,36], has a significant impact on prognosis [37] and response to treatment.

Macrophages are divided into types M1 and M2. Anti-inflammatory macrophages, called the “M2 type”, as opposed to M1 pro-inflammatory macrophages, promote an immunosuppressive environment through their high expression of cytokines such as IL-10 and transforming growth factor β (TGFβ) [9].

**Figure 2:**
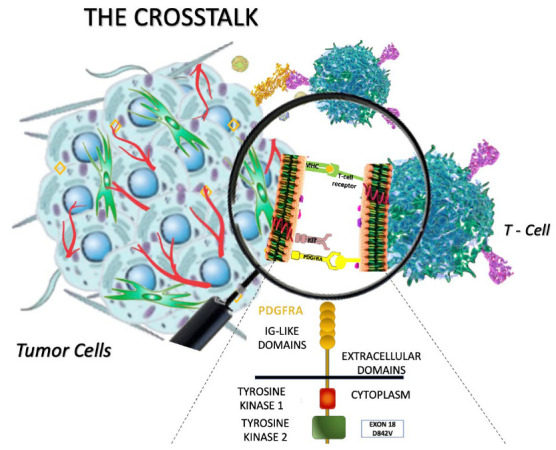
The crosstalk between tumor cells and T lymphocytes in GIST. PDGFRA-mutant GISTs, compared to KIT-mutant GISTs, show an increased number of immune cells (such as NK+, CD4+, and CD8+ lymphocytes) and higher production of immunogenic neo-antigens, leading to increased tumor regression (based on Vitiello et al.) [5].

The crosstalk between tumor cells and T lymphocytes is shown in Figure 2.

M2 macrophages are implicated in the promotion of neoplastic spreading through the stimulation of angiogenesis, the proliferation of cancer cells, and the remodeling of extracellular matrix (ECM); in fact, this subtype of TAMs is more expressed in metastatic lesions than in primary GISTs [7,38,39]. Furthermore, metastases are enriched by a high number of TILs, including CD8+ cytotoxic T lymphocytes (CTLs), CD4+ T helper type 1 lymphocytes (Th1), CD4+ T helper type 2 lymphocytes (Th2), IL-17+ T helper cells (Th17), and Tregs [36,40,41].

M2 macrophages and Tregs are the most represented cells, and they determine a strongly immunosuppressive TME in GISTs [7].

A small fraction of tumor-infiltrating immune cells is represented by B cells and DCs, which are usually poorly expressed or even absent in GISTs [42].

NK cells are innate immune system lymphocytes involved in immune response to tumors, and they seem to be interestingly enriched in the GIST microenvironment where they specifically target cells with a lower expression level of major histocompatibility complex 1 (MHC I), a common feature of these neoplasms. Rusakiewicz et al. [37] demonstrated that the number of NK cells, localized mainly within the fibrous stroma surrounding tumor cells [36], was significantly associated with low mitotic index in a cohort of 91 GIST patients [9]. A high level of CD3+ T and NK cells correlated with a reduced relapse rate and a more favorable prognosis in untreated metastatic GISTs [9,36,37].

CD3+ T and B cells are more concentrated in intestinal and highly proliferating GISTs as compared with those arising in the stomach and with a low proliferation index (<10%) [36]. Even though the knowledge about the TME is growing, the correlations between immune cells and other prognostic factors (tumor location, size, or mitotic index) are still controversial [43], suggesting that other factors may influence the composition of the TME.

### 3.2. Looking Forward: Driver Mutations and Immune Microenvironment

The type of GIST driver mutation represents an important prognostic factor, correlating with clinical features and biological aspects of the disease [5]. The presence of deletions within c-KIT codons 557/558 is associated with a more aggressive behavior compared to other exon 11 mutations, thus resulting in shorter recurrence-free survival (RFS) for patients with resected GIST and shorter PFS for metastatic patients [44,45].

Recent evidence also suggests that the genotype can influence immune cells infiltrating the TME [43]. Surprisingly, PDGFRA-mutant GISTs showed an increased number of immune cells, compared with c-KIT-mutant GIST, and an overexpression of stimulatory cytokines (e.g., CXCL14) which additionally activate NK+, CD4+, and CD8+ cells, leading to tumor regression [5]. In particular, a study by Xiangfei S. et al. [10] demonstrated that TILs were more abundant in GISTs harboring a PDGFRA mutation. Another study by Vitiello et al. [5] showed that 75 KIT-mutant GIST patients harbored a lower number of immune cells than did PDGFRA-mutant GISTs.

Gasparotto et al. [43] studied the possible correlation between the presence and type of driver mutation and neo-antigens’ immunogenic capability to bind to patient-specific HLA types. Tumor neo-antigens are short peptides that can interact with HLA molecules and be presented on the surface of tumor cells to activate T-cells’ cytotoxic immunity. It turned out that GISTs carrying KIT and PDGFRA driver mutations produced more immunogenic neo-antigens compared to BRAF- or NF1-mutated GISTs and harbored a richer immune infiltrate [43]. WNT/β-catenin signaling (WNT/β-cat), RAS, and the Hedgehog (HH) pathway, usually activated in K/P WT GIST, could lead to lower tumor immune infiltration and immune evasion [43].

In 38 primary PDGFRA-mutant GISTs, greater numbers of neoepitopes and suppressor cells were found [43].

Not only the gene but also the type of pathogenic variant (PV) can relate to different features. D842V-mutated GISTs are more enriched in immune cells, mainly CD8+ T cells, than are non-D842V ones [10,46].

These findings suggest that PDGFRA-mutant GISTs, characterized by intrinsic resistance to standard TKIs and a better prognosis, are more immunogenic compared to genetic alterations sensitizing to common TKIs [5].

### 3.3. Driver Mutations and Immune Checkpoint Expression to Improve Prognostication in GIST

The expression of immune checkpoints and its relationship to the clinical phenotype in GIST is not understood, as it has previously been poorly evaluated.

Immune checkpoints, such as PD-L1, PD-1, and CTLA-4, by escaping immune surveillance, play a key role in tumor progression and influence the survival of patients with solid tumors [16,47]. According to the recent knowledge, despite some known limitations, immune checkpoint expression could be a potential prognostic factor and predictive biomarker of response to immune checkpoint inhibitors in patients with solid tumors [45,48,49,50,51].

PD-1, a type I transmembrane protein of the immunoglobulin superfamily, and its ligands are key regulators in a wide spectrum of immune responses and play a critical role in autoimmunity and self-tolerance, as well as in cancer immunology [52]. PD-1 is expressed on a variety of immune cells, such as monocytes, T cells, B cells, DCs, and TILs, while PD-L1, the main ligand for PD-1, is expressed on several hematopoietic cells, especially on tumor antigen-presenting cells (APCs), and on peripheral nonhemopoietic cells. [53]. Moreover, PD-L1 can be highly expressed on DCs or on the tumor cells themselves [54]. PD-L2 is expressed on APCs and other immune and non-immune cells [53]. The PD1/PD-L1 axis is the most common immune checkpoint pathway, and it impairs T-cell proliferation and effector functions, leading to apoptosis of tumor-specific T cells [55]. Several results from the literature have shown that high expression of PD-L1 is related to poor clinical outcomes in patients with solid tumors. Previous findings showed that tumor PD-L1 expression, evaluated via immunohistochemistry (IHC) on formalin-fixed and paraffin-embedded tumor sections, was greater in GISTs than in several types of soft tissue sarcomas [56], and the PD-L1 expression level was also associated with high-risk GIST patients showing poorer outcomes in therapeutic settings [57].

Recently, circulating immune checkpoint molecules have been shown to have potential prognostic significance in metastatic GISTs [58]. High levels of plasma PD-1, PD-L1, and the butyrophilin family proteins sBTN3A1 and pan-BTN3As seem to predict a shorter PFS and a poor prognosis in patients with KIT exon11-mutated metastatic GIST treated with IM as a first-line treatment [56]. Interestingly, in the same study, lower plasma levels of soluble PD-L1 and pan-BTN3As and the absence of KIT exon 11 deletions or deletions/insertions at codons 557 and/or 558 were significant prognostic factors for a longer PFS in mGIST patients, showing different expression profiles of immune checkpoints in GISTs harboring different driver mutations [58].

Preliminary data highlight the potential role of PD-L1 expression as an independent prognostic factor also in PDGFRA-mutant GISTs [10]. The expression of PD-L1 is heterogeneous in PDGFRA-mutant GISTs, and it is inversely related to tumor size, suggesting the inhibition of tumor proliferation and a better prognosis [10]. Furthermore, PDGFRA-driven GISTs express a high concentration of indoleamine-2,3-dioxygenase (IDO), an immune checkpoint molecule that is correlated with high inflammatory cell infiltrates, CD4+ cells in particular [10].

In a recent study, Seifert et al. [59] analyzed matched tumor and blood samples from 85 patients with GIST and studied the expression of immune checkpoint molecules other than PD-1/PD-L1 using flow cytometry. Seifert et al. [59] found that immune checkpoint molecules such as lymphocyte activation gene 3 (LAG3) and T-cell immunoglobulin mucin 3 (TIM3) are upregulated on TILs in GIST tissue.

The relationship between prognosis and the quality of stromal differentiation (SD) and immune checkpoint expression in GIST has been investigated, with possible clinical practice implications for SD in the near future as a prognostic tool.

In immune checkpoint inhibitor analysis, authors focused on PD-L1 and v-domain Ig suppressor of T-cell activation (VISTA) levels, the latter being a biomarker of the tumoral microenvironment status.

Of note, an immature stroma was found to be associated with lower PD-L1 expression and VISTA, as well as a more aggressive phenotype (higher disease stage, higher tumoral grade, and higher mitosis). Like in other studies, PD-L1 expression confirmed a poorer prognostic significance, whereas VISTA positivity in immune cells was found to be protective [56].

The links between immune checkpoints, especially PD-L1 expression, the TME, and the clinical behavior of GISTs with different driver mutations are under investigation and still represent an intriguing research field.

## 4. Impact of Immune Microenvironment on Treatment Approach in GIST

### 4.1. Beyond the Tumor: The Immune-Modulating Effects of Imatinib

**Figure 3:**
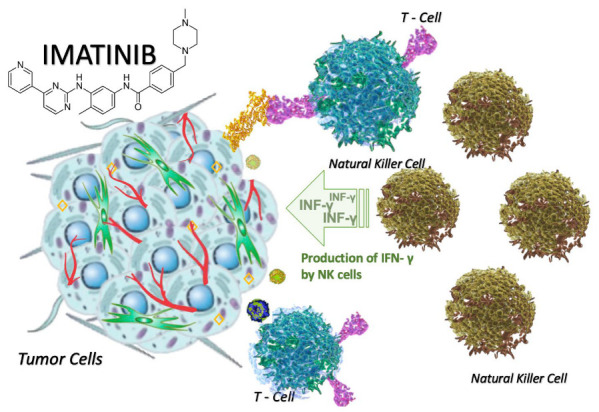
Modification in the tumor microenvironment after the use of imatinib. IM increases the number of NK cells but also supports KIT-dependent crosstalk between DCs and NK cells, resulting in increased production of immune-stimulating IFN-γ. In summary, the inhibition of c-KIT decreases immune-suppressor cells and enhances antitumor immune function [60,61].

Along with the direct inhibition of oncogenic signaling, IM can modulate tumor-infiltrating immune cells, enhancing an immuno-stimulatory microenvironment through different mechanisms [15] (Figure 3).

The immune-stimulating effect of IM is mediated by a reduction in the level of IDO, an immune checkpoint molecule able to inhibit T cells [60].

Through IDO blockade, IM increases the number of intratumor CD8+ T cells and reduces Tregs, leading to stimulation of the immune response against tumor cells [16,62].

c*-KIT* is also expressed on the surface of immune cells (i.e., mast cells), and its pathway, usually upregulated by activating driver mutation, plays an important role in the recruitment of innate immune cells (DCs, NK, CD8+, and CD4+ T cells) and the regulation of immune-suppressive cells (Tregs) [63].

Several recent lines of evidence suggest direct correlations between the TIL counts in cancer tissue, the ratio of CD8+ effector T cells to Tregs, and a favorable prognosis in various malignancies [64,65].

Inhibition of *KIT* signaling by IM is important for the downregulation of IDO. In fact, high levels of IDO and a low ratio of CD8+/Treg cells have been correlated to primary or acquired resistance to target agents [16,37]. The inhibition of c-KIT with TKIs alone or associated with specific antibodies can decrease the number of immune-suppressor cells and enhance antitumor immune function [60,61].

Based on this evidence, an open-label, multi-center, single-arm, phase 2 trial (NCT03291054), started in 2017, is testing the overall response rate (ORR) of patients with advanced GIST treated with epacadostat, an IDO inhibitor, in combination with pembrolizumab after failing on at least two TKI regimens.

IM additionally supports *KIT*-dependent crosstalk between DCs and NK cells, resulting in the production of immune-stimulating interferon-gamma (IFN-γ). Enhanced IFN-γ production by NK cells has been reported in patients affected by metastatic GIST after IM treatment. Patients with a high number of activated NK cells after IM therapy have a good prognosis [36] and can be identified as “immunologic responders” because of their better response to cytokine-based immunotherapy [37,66]. In a mouse model, Kats et al. proved an increasing production of INF-γ and a reduction in tumor size after treatment with anti-KIT CAR T cells [67].

Cytotoxic T-lymphocyte-associated antigen 4 (CTLA4) is an inhibitory immune-checkpoint receptor expressed on the surface of activated CD8+ T cells and on CD4+ T cells, which are implicated in the down-modulation of T helper and upregulation of Treg immune-suppressive activity [47]. Preclinical data have shown that CTLA-4 blockade in GIST-bearing mice can lead to the accumulation of CD8+T cells with enhanced INF-γ production [68].

This mechanism may explain the beneficial effect of combining IM with CTLA-4 blockade, observed by Balachandran et al. [60]. CTLA-4 blockade synergizes with TKIs in mouse models, leading to the study of this combination in humans.

In a clinical trial, all eight patients with a stage III/IV GIST treated with combined INF-α and IM achieved a complete response (CR) [69]. Before the administration of INF-α and IM, INF-γ was barely detectable; after 4 weeks of treatment, TILs increased in number and produced a high level of INF-γ [69].

### 4.2. The Clinical Relevance of the Multifaceted Role of the Immune System: Immunotherapy for GIST Patients?

The recent introduction of immunotherapy, with the approval of immune checkpoint inhibitors (ICIs) targeting PD-1 and PD-L1, has revolutionized the treatment of several cancer types [70,71,72] and improved the survival rates of patients [17,73,74,75,76,77,78,79,80].

Immune checkpoint inhibitors (such as anti-CTLA4, anti-PD-1/PD-L1, anti-TIM-3, or anti-LAG3 antibodies) and IDO inhibitors could become a potential future strategy to improve the effects of targeted therapy in GIST [16,17]. In fact, despite the efficacy of tyrosine kinase inhibition, patients with metastatic GIST develop resistance to target therapy and tumor progression. To date, knowledge of the efficacy of immunotherapy in this setting is limited, and few clinical trials were designed (Table 1) during the last decade.

### 4.3. Combination Therapy with TKIs and ICIs

The study of immunotherapy in GISTs is evolving. In the randomized unblinded phase II trial NCT02880020, patients with advanced/metastatic refractory GIST were enrolled and randomized 1:1 to receive either nivolumab (240 mg Q2 wks) or nivolumab (240 mg Q2 wks) plus ipilimumab (1 mg/kg Q6 wks) for up to 2 years. In the nivolumab arm, 7/15 (46.7%) patients had stable disease (SD) with a median PFS of 8 weeks, while in the nivolumab + ipilimumab arm, 2/12 (16.7%) had SD, and the median PFS was 9 weeks [81].

In a phase I trial (NCT01738139), no responses were seen among 35 KIT-mutant GIST patients treated with the combination of ipilimumab (3 mg/kg) and IM (400 mg orally twice daily). Only one patient with a wild-type gastric GIST showed stable disease and continued to receive the treatment for 16 months [82]. In a phase I study (NCT01643278), 20 patients with unresectable/metastatic GIST were enrolled and received ipilimumab plus dasatinib. All patients had primary or secondary KIT resistance mutations or primary PDGFRA mutations. Most patients featured rapid disease progression according to the RECIST criteria, and few (7/13) had partial responses according to the Choi criteria, with a median PFS of less than 3 months. Only one patient, whose GIST harbored a *PDGFRA* exon 18 D842V mutation, remained in the trial for about 13.9 months [83].

The phase 1b/2 trial NCT03609424 is studying the efficacy of IM plus PDR001 (spartalizumab), an anti-PD-1 antibody, in advanced GIST after the failure of standard TKI therapies including IM, sunitinib, and regorafenib.

Two clinical trials are evaluating the efficacy of avelumab in GISTs. In REGOMUNE (NCT03475953), avelumab is administered in association with regorafenib in multiple solid tumors. The AXAGIST study (NCT04258956), a phase II single-arm trial, is testing the antitumor activity of avelumab in combination with axitinib in patients with unresectable/metastatic GIST after progression on second- or third-line treatment.

Hopefully, the results of these ongoing trials will soon provide new treatment frontiers.

## 5. Conclusions

Tumor mutational status is biologically and clinically important in many types of tumors [84] and has made GIST a paradigmatic model of oncogene addiction. GISTs are composed of many different genetic subtypes. Despite the relevance of mutational status, GISTs represent a heterogeneous genetic and clinical subgroup, showing variable clinical outcomes even in patients showing the same KIT or PDGFRA mutation.

The current research paradigm in oncology is shifting to the immune system and the TME [85], and recent literature data highlighted the potential role of the TME in GISTs as well, beyond the known impact of the mutational status. Therefore, deciphering the key activities of tumoral microenvironment components, and how they are influenced by each other, may be the answer to clinical heterogeneity in GIST, going further than the known paradigm model of oncogene addiction.

## Figures and Tables

**Table 1:** Clinical Trials of Immunotherapy in GISTs.

Year	Title	Trial Phase	Primary End-Point	ClinicalTrials.Gov Identifier
2012	Phase I Study of Dasatinib in Combination With Ipilimumab for Patients With Advanced Gastrointestinal Stromal Tumor and Other Sarcomas	I	Maximum tolerated dose (MTD)	NCT01643278 Completed
2012	A Phase I Trial of Ipilimumab (Immunotherapy) and Imatinib Mesylate (c-Kit Inhibitor) in Patients With Advanced Malignancies	I	MTD	NCT01738139 Recruiting
2015	Nivolumab With or Without Ipilimumab in Treating Patients With Metastatic Sarcoma That Cannot Be Removed by Surgery	II	Overall response rate (ORR)	NCT02500797 Active, not recruiting
2016	A Randomized Phase 2 Study of Nivolumab Monotherapy Versus Nivolumab Combined With Ipilimumab in Patients With Metastatic or Unresectable Gastrointestinal Stromal Tumor (GIST)	II	ORR	NCT02880020 Completed
2017	A Phase II Study of Epacadostat and Pembrolizumab in Patients With Imatinib Refractory Advanced Gastrointestinal Stromal Tumors	II	ORR	NCT03291054 Completed
2018	A Phase Ib/II Study of PDR001 Plus Imatinib for Metastatic or Unresectable GIST With Prior Failure of Imatinib, Sunitinib and Regorafenib	I/II	Maximum tolerated dose; Disease control rate	NCT03609424 Recruiting
2018	A Phase 1 Multiple Dose Study to Evaluate the Safety and Tolerability of XmAb^®^18087 in Subjects With Advanced Neuroendocrine and Gastrointestinal Stromal Tumors (DUET-1)	I	Safety and tolerability profile; MTD	NCT03411915 Completed
2018	A Phase I/II Study of Regorafenib Plus Avelumab in Solid Tumors (REGOMUNE)	I/II	Maximum tolerated dose; Disease control rate	NCT03475953 Recruiting
2019	Phase Ib Study of TNO155 in Combination With Spartalizumab or Ribociclib in Selected Malignancies	Ib	Response (CR or PR)	NCT04000529 recruiting
2020	A Phase II, Single Arm Study of Avelumab In Combination With Axitinib in Patients With Unresectable/Metastatic Gastrointestinal Stromal Tumor After Failure of Standard Therapy—AXAGIST	II	3-Month Progression-Free Survival (PFSR) Rate	NCT04258956 Recruiting

## Data Availability

Not applicable.
